# Endosonography-guided choledochoduodenostomy using a lumen apposing metal stent vs. ERCP: Individual patient data and aggregate meta-analyses

**DOI:** 10.1055/a-2863-1621

**Published:** 2026-06-01

**Authors:** Yen-I Chen, Marco Spadaccini, Anand Sahai, Bertrand Napoleon, Gianfranco Donatelli, Andrea Anderloni, Rastislav Kunda, Cesare Hassan, Myriam Martel, Carlo Fabbri, Sharif Yassin, Shannon Melissa Chan, Paolo Giorgio Arcidiacono, Alessandro Fugazza, Eric Lam, Pradermchai Kongkam, Nauzer Forbes, Alberto Larghi, Jeffrey Mosko, Schalk Van der Merwe, Seng Ian Gan, Romain Legros, Jeremie Jacques, Avijit Chatterjee, Thawee Ratanachu-ek, Corey Miller, Payal Saxena, Etienne Desilets, Gurpal Sandha, Yeganeh R. Moghaddam, Alan Barkun, Alessandro Repici, Anthony Y.B. Teoh

**Affiliations:** 1 Division of Gastroenterology and Hepatology 54473 McGill University Health CentreMontrealCanada; 2 Division of Gastroenterology and Digestive Endoscopy 9268 IRCCS Humanitas Research HospitalRozzanoLombardiaItaly; 3 Pieve EmanueleDepartment of Biomedical Sciences 437807 Humanitas UniversityMilanItaly; 4 Gastroenterology Department 25443 Centre Hospitalier de l'Universite de MontrealMontrealCanada; 5 Endoscopy unit 89686 Jean Mermoz Private HospitalLyonAuvergne-Rhône-AlpesFrance; 6 Unité d'Endoscopie Thérapeutique 55727 Ramsay Santé Hôpital privé des PeupliersParisÎle-de-FranceFrance; 7 Departement of Clinical Medicine and Surgery 9307 University of Naples Federico IINapoliItaly; 8 Digestive Endoscopy Unit, Division of Gastroenterology 18631 Fondazione IRCCS Policlinico San MatteoPaviaLombardiaItaly; 9 Department of Surgery 60201 Universitair Ziekenhuis BrusselBrusselBelgium; 10 Digestive Endoscopy and Gastroenterology Unit, Forlì-Cesena Hospitals 390233 Azienda Unita Sanitaria Locale della RomagnaForlì-CesenaItaly; 11 Surgery 13621 Prince of Wales HospitalHong KongHong Kong; 12 Pancreatobiliary Endoscopy and Endosonography 9372 IRCCS Ospedale San RaffaeleMilanLombardyItaly; 13 Gastroenterology 8156 St Paul's HospitalVancouverCanada; 14 Medicine 26683 Chulalongkorn UniversityBangkokThailand; 15 Medicine 2129 University of CalgaryCalgaryCanada; 16 Digestive Endoscopy Unit 18654 Fondazione Policlinico Universitario Agostino Gemelli IRCCSRomeLazioItaly; 17 Gastroenterology 10071 St Michael's HospitalTorontoCanada; 18 Department of Gastroenterology and Hepatology 60182 University Hospitals LeuvenLeuvenFlandersBelgium; 19 Department of Gastroenterology 8167 Vancouver General HospitalVancouverCanada; 20 Service d'hépato-gastro-entérologie 558112 University Hospital Centre of Limoges Dupuytren 2LimogesAquitaine-Limousin-Poitou-CharentesFrance; 21 Faculty of Medicine 12365 University of OttawaOttawaCanada; 22 Medicine 10055 Ottawa Hospital Research InstituteOttawaCanada; 23 Department of Surgery 54781 Rajavithi HospitalBangkokBangkokThailand; 24 Department of Gastroenterology 2205 Royal Prince Alfred HospitalCamperdownNew South WalesAustralia; 25 Gastroenterology 10023 Hôpital Charles-LeMoyneGreenfield ParkCanada; 26 Internal Medicine 25484 University of Alberta HospitalEdmontonCanada; 27 Surgery 13620 Hong Kong Sanatorium & Hospital LimitedHong KongHong Kong

**Keywords:** Pancreatobiliary (ERCP/PTCD), Endoscopic ultrasonography, Biliary tract, Intervention EUS

## Abstract

**Background and study aims:**

Endoscopic ultrasound-guided choledochoduodenostomy using a lumen-apposing metal stent (EUS-CDSL) has been shown in randomized controlled trials (RCTs) to be effective compared with endoscopic retrograde cholangiopancreatography (ERCP) in patients with advanced malignant distal biliary obstruction (MDBO). We aimed to further ascertain safety of EUS-CDSL with greater statistical power.

**Patients and methods:**

We undertook individual patient data (IPD) and aggregate meta-analyses following the PRISMA-IPD statement. A literature search was performed from January 2013 to November 2024 using OVID MEDLINE, EMBASE, Cochrane Library, and ISI Web of Science. Additional searches were performed for abstracts and the gray literature. RCTs comparing EUS-CDSL with ERCP were included. The primary outcome was odds of 30-day procedure related adverse events (AEs). Secondary outcomes included procedure-related 14-day severe or fatal AEs, technical success, clinical success, stent dysfunction, and procedure time.

**Results:**

A total of 2679 citations were screened with three RCTs included (519 patients). The odds ratios (ORs) for 30-day AEs between EUS-CDSL and ERCP were similar (OR 0.72, 95% confidence interval [CI] 0.44–1.16). There was also no significant difference in severe or fatal AEs (OR 0.64, 95% CI 0.22–1.83). The ORs for technical success were greater for EUS-CDSL (OR 4.22, 95% CI 2.35–7.61) with shorter procedure time (mean difference -10.50 minutes (95% CI -15.28 to -5.73). No significant differences were noted in clinical success or stent dysfunction.

**Conclusions:**

Our IPD meta-analysis demonstrates safety of EUS-CDSL as a first-line alternative to ERCP in MDBO and dilated biliary tree. In addition, EUS-CDSL is associated with higher technical success and shorter procedure time.

## Introduction

**Fig. 1:**
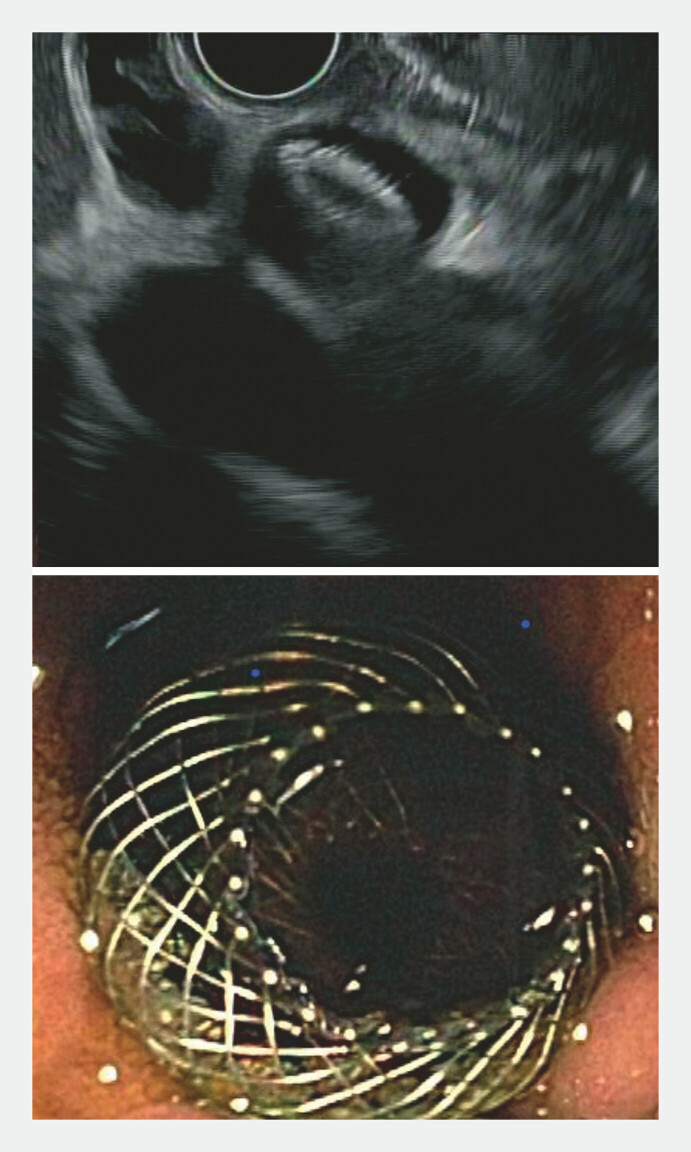
EUS-guided choledochoduodenostomy using a lumen apposing metal stent.

Malignant distal biliary obstruction (MDBO) leading to painless jaundice is the most common presentation of peri-ampullary cancers 1 . Restoring bile flow has been associated with increased survival and is essential in alleviating symptoms of cholestasis, improving patient quality of life, and allowing for chemotherapy administration 2 3 . Endoscopic retrograde cholangiopancreatography (ERCP) has been the standard of care for relieving MDBO for over four decades 4 . ERCP, however, remains a challenging procedure with up to 10% to 20% technical failure in MDBO along with being associated with significant risk for complications such as post-ERCP pancreatitis 4 5 6 . Endoscopic ultrasound-guided choledochoduodenostomy (EUS-CDS) using a lumen apposing metal stent (LAMS) (EUS-CDSL) is an emerging modality, which achieves biliary drainage through creation of an anastomosis between the extra-hepatic bile duct and the duodenum proximal to the ampulla. The advent of the cautery-assisted LAMS technique has greatly simplified EUS-CDSL, allowing for direct stent insertion without need for a separate access device, wire guidance, and/or tract dilation ( Fig. 1 ) 7 8 9 . Two recent randomized controlled trials (RCTs) comparing EUS-CDSL vs. ERCP in MDBO have demonstrated comparable or better technical success and shorter procedure time for EUS-CDSL, without significant differences in stent patency and adverse events (AEs) 6 10 . These multicenter RCTs have provided high-quality data supporting an alternative first-line modality to ERCP. Like most interventional trials, however, these studies were not sufficiently powered to adequately assess comparability in terms of AEs. As EUS-CDSL gains clinical adoption given its technical ease, especially considering recent RCTs data supporting its use, it is imperative to adequately assess its safety while also better characterizing other important clinical outcomes.

The primary research question of our individual patient data (IPD) and aggregate meta-analyses is whether EUS-CDSL is comparable in safety to ERCP as a first-line approach in patients with MDBO. We hypothesized that EUS-CDSL is akin in safety to ERCP. In addition, we postulated that EUS-CDSL is superior in technical efficiency and success while being comparable with regards to stent function.

## Methods

### Overview

We performed a standard aggregate systematic review and meta-analysis following the Preferred Reporting Items for Systematic Review and Meta-Analysis (PRIMSA) reporting standards 11 . An IPD meta-analysis was also undertaken according to the PRISMA-IPD statement 12 . Our study was registered with the International Prospective Register of Systematic Reviews (CRD 485070) and was approved by the research ethics board at all coordinating sites. Our primary outcome was 30-day risk for procedure-related AEs. Secondary outcomes included the 14-day risk for severe or fatal procedure-related AEs, technical success, clinical success, stent dysfunction, and procedure time.

### Search strategy and study selection

A comprehensive literature search was initially performed from January 2013 to November2023 and was subsequently updated to November 2024. The search was conducted using OVID MEDLINE, EMBASE, Cochrane Library, and ISI Web of Science with MeSH and controlled vocabulary for terms specified for: 1) endoscopic ultrasound guided biliary drainage; 2) endoscopic ultrasound guided choledochoduodenostomy; 3) endosonography-guided biliary drainage; and 4) endosonography-guided choledochoduodenostomy (Supplementary file 1). Two reviewers (YR and SY) performed title and abstract screens of every identified citation. A third reviewer was involved for conflict resolution (YC). A comprehensive search of grey literature sources was also conducted, including conference abstracts, trial registries, theses, and relevant non-peer-reviewed materials, to ensure the inclusion of unpublished or hard-to-access studies. Cross-referencing of identified studies was performed. Citations were handled using Endnotes 20.5 (Clarivate, Philadelphia, Pennsylvania, United States)

### Eligibility

A study was included if it met all the following inclusion criteria: The study was a RCT involving adult patients with MDBO, the experimental arm was EUS-CDS using a LAMS as a first-line modality, and the control arm was ERCP with transpapillary self-expanding metal stent insertion. Studies assessing EUS-CDS using other types of stents other than biliary LAMS were excluded.

### Data items

A data extraction form was created to collate all data from all studies using unified definitions and scales. Data items included: age, sex, American Society of Anesthesiologists classification, etiology of MDBO, tumor stage, and baseline serum bilirubin level and clinical outcomes and their parameters. Data were collected and reviewed locally by the principal investigator of the respective trials (YC, MS, and AYBT) then submitted for central review (YC and MM).

### IPD integrity

Following identification of eligible studies, the corresponding authors of each trial were contacted for patient-level data. Sharing of deidentified data occurred only following research ethics approval at each of the included RCT principal investigator sites. Prior to analysis, information was examined centrally for missing data, harmonization of variable coding and definitions across studies, and verification of outcome results with published report. Baseline characteristics were reviewed for all trials to identify potential imbalances.

### Risk of bias

Study risk of bias was assessed using the revised risk of bias (ROB 2), as recommended by the Cochrane Handbook 13 , with the ROBVIS (Risk-Of-Bias VISualization) tool to create risk-of-bias plots 14 . Two experienced reviewers were involved in assessment of bias (YC and MM). Certainty of evidence was determined using the Grading of Recommendations Assessment, Development and Evaluation (GRADE) framework 15 . Given the lack of grading system for IPD, the GRADE was performed solely on results of the aggregated meta-analysis. Certainty of evidence for each selected outcome of interest was rated as high, moderate, low, or very low, using the GRADEpro GDT software (Evidence Prime Inc., Hamilton, Ontario, Canada).

### Outcomes

The primary outcome was overall 30-day procedure-related AEs as graded by American Society for Gastrointestinal Endoscopy (ASGE) lexicon for endoscopic AEs 16 . Secondary endpoints included the 14-day procedure-related severe or fatal AEs (ASGE lexicon), technical success, procedure time, clinical success, and stent dysfunction. Technical success was defined as successful insertion of a transpapillary or choledochoduodenostomy stent at the index procedure. Procedure time was defined as time from scope insertion to scope withdrawal. Clinical success was defined as a 50% decrease in bilirubin within 2 weeks post-stent insertion or achieving a value < 25% of pre-procedure bilirubin levels within 4 weeks post stent insertion 5 17 18 . Stent dysfunction was defined as endoscopic or radiologic reintervention confirming stent blockage or migration needing stent cleaning, stent change, and/or additional stent insertion, and at least one of the following: 1) suspected cholangitis (Tokyo consensus definition 19 ); 2) definite cholangitis (Tokyo definition 19 ); 3) ≥ 50% increase in bilirubin from the lowest level post index procedure; or 4) ≥ 20% increase in bilirubin from the lowest level post index procedure as well as evidence of obstruction on imaging 10 . Patients with a bilirubin that never decreased post index stenting were not classified as experiencing stent dysfunction but, rather, were categorized as having failed to achieve initial clinical success. Notably, outcomes were categorized as primary and secondary according to a prespecified analytic hierarchy. In the context of a IPD meta-analysis, this classification reflects the relative importance of endpoints rather than a confirmatory hypothesis-testing framework.

### Statistical analysis

Both aggregate and IPD meta-analyses were performed as described below. IPD results were interpreted using standardized definitions for each endpoint across both trials with all analyses based on an intention-to-treat principle. A one‐stage approach was adopted a priori because this method uses a more exact statistical methodology than normal approximation and is the preferred technique when only few studies are available 20 . We used PROC GLIMMIX in SAS (SAS Institute Inc., Cary, North Carolina, United States), a generalized logistic mixed effects model with random intercepts for study/center to estimate adjusted odds ratios (ORs) and 95% confidence intervals (CIs) for all endpoints. If a limited number of studies or insufficient variation in the response led to convergence issues, alternative models were explored by incorporating study/center as a fixed effect within a multiple regression model, If the issue persisted, the random intercept was removed to ensure model stability. Two-sided *P* ≤ 0.05 was considered evidence of statistical significance.

For the aggregate meta-analysis, effect size was calculated with mean differences for continuous variables and risk ratios (RRs) for categorical variables. The DerSimonian and Laird method for random effect models was applied to all outcomes to determine corresponding overall effect sizes and their CIs. Sensitivity analyses were performed using the fixed effects models when no statistical heterogeneity was noted. Mean differences were handled as continuous variables using the inverse variance approach. The Higgins I ^2^ statistic and the between-study variance (τ²) were calculated to quantify the proportion of variation in intervention effects attributable to between-study heterogeneity. Values of 0% to 40%, 30% to 60%, 50% to 90%, and 75% to 100% for the I² statistic represent a potential of low, moderate, substantial, and considerable heterogeneity, respectively, and were interpreted with size and direction of effect as well as strength of evidence of heterogeneity using a Chi-square test of homogeneity with a 0.10 significance level 21 . Analyses were done using Meta package in R version 2.13.0 (R Foundation for Statistical Computing, Vienna, Austria, 2008).

## Results

**Fig. 2:**
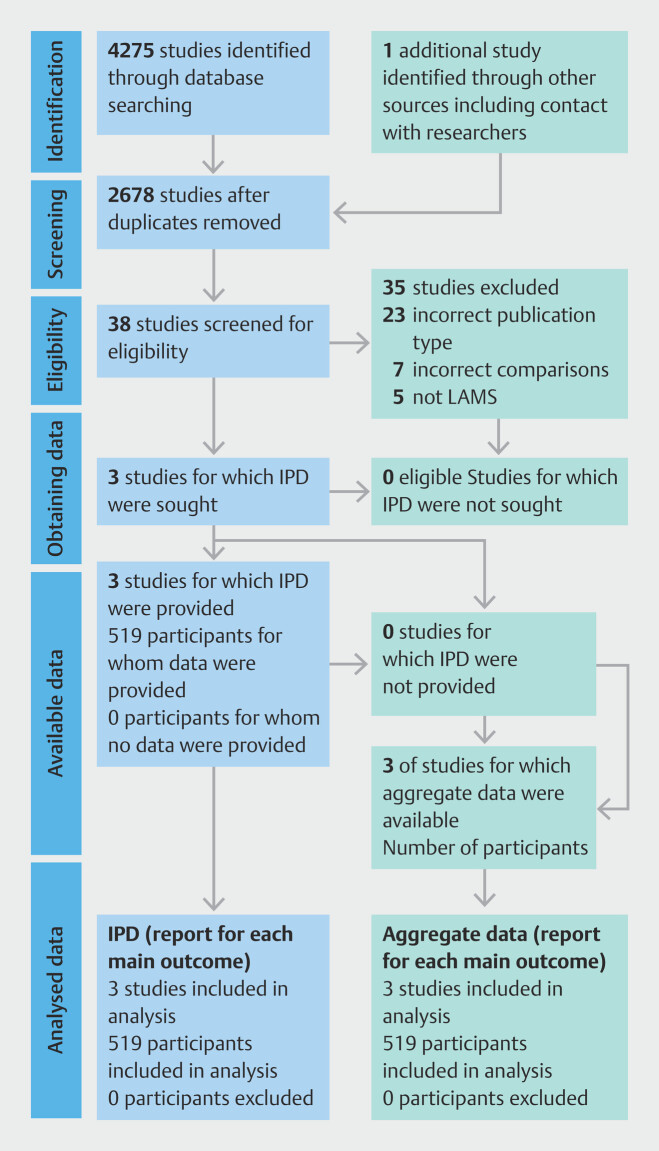
PRISMA IPD flow diagram 12 .

A total of 2679 citations were identified by the literature searches. Ultimately, three RCTs 6 10 21 (n = 519 patients) were selected for analysis using the predefined eligibility criteria ( Fig. 2 ). The first author from each RCT provided anonymized individual patient-level data from their respective trials. IPD was available for all 519 patients. No issues were noted with any of the individual datasets. Lack of variation for stent dysfunction and technical success prevented a hierarchical-level analysis by studies and therefore results are reported using unadjusted ORs.

**Fig. 3:**
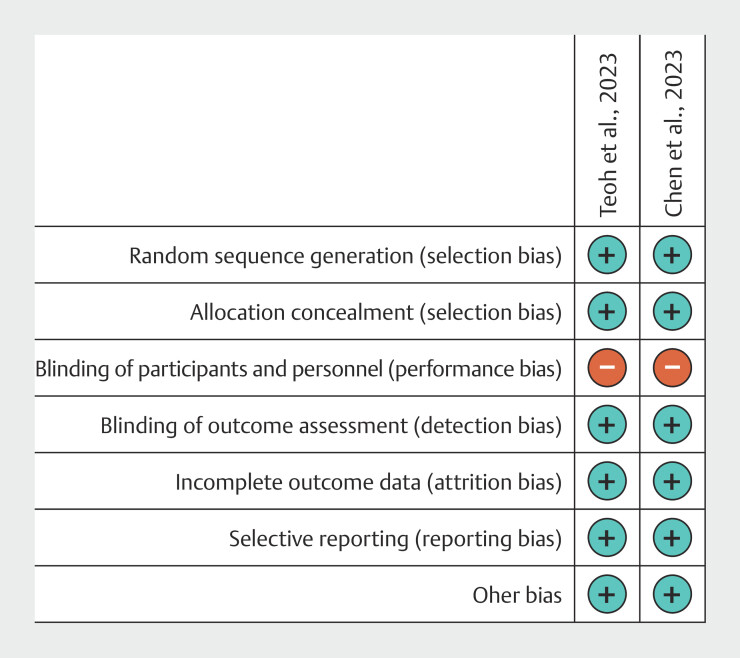
Cochrane risk of bias.

Of the total 519 patients receiving biliary drainage, 263 were allocated to EUS-CDSL and 256 to ERCP. All trials were multicenter with broad geographical representation, including centers from Canada, China, France, Belgium, Italy, the Netherlands, Australia, and Thailand. Participating endoscopists could not be blinded due to the nature of the intervention, which may have resulted in risk of operator bias; however, there were no deviations from the intended intervention, leading to a RoB2 low risk of bias ( Fig. 3 ). Grading of the evidence based on aggregate data was moderate for all endpoints except procedure time, which was graded low due to statistical heterogeneity and wide CIs (Supplementary file 2). No significant heterogeneity was noted in the aggregate meta-analysis except for procedure time, which exhibited substantial heterogeneity ( *P* = 0.07; I ^2^ = 69%, τ ^2^ = 12).

Patient demographics from pooled IPD across the three trials are summarized in Table 1 . Using IPD, pooled rates of 30-day AEs were 12.9% (34/263) and 17.2% (44/256) for EUS-CDSL and ERCP, respectively ( *P* = 0.17). The pooled rates for severe or fatal procedure-related AEs were 2.3% (6/263) for EUS-CDSL and 3.5% (9/256) for ERCP ( *P* = 0.33). The pooled rate for technical success was 93.9% for EUS-CDSL and 78.5% for ERCP ( *P* < 0.01) with mean procedure time of 15.1 ± 13.9 minutes and 25.6 ± 16.5 minutes, respectively ( *P* < 0.01). There were no significant differences in clinical success or stent dysfunction ( Table 2 ).

**Table:** **Table 1** Pooled patient demographics using individual patient data.

**Variable**	**Overall n = 519**	**EUS-CDS n = 263**	**ERCP n = 256**	***P* value **
Age (years)	73.0 ± 11.0	74.0 ± 10.8	71.9 ± 11.1	0.03
Female	229 (44.1%)	103 (40.2%)	125 (47.9%)	0.08
ASA				
I-II	357 (68.8%)	172 (33.2%)	185 (35.7%)	0.09
III-IV	162 (31.2%)	91 (34.6%)	71 (13.7%)	
Etiology of MDBO				
Pancreatic cancer	468 (90.2%)	234 (89.0%)	234 (91.4%)	0.35
Cholangiocarcinoma/gallbladder cancer	23 (4.4%)	14 (5.3%)	9 (3.5%)	0.32
Ampullary cancer	11 (2.1%)	6 (2.3%)	5 (2.0%)	0.80
Other	17 (3.3%)	9 (3.4%)	8 (3.1%)	0.85
Tumor stage (data available for 2 sites)				
Borderline/locally advanced	243 (81.3%)	31 (20.4%)	25 (17.1%)	0.45
Unresectable	56 (18.7%)	121 (79.6%)	122 (83.0%)	
Baseline bilirubin level (µmol/L)	239.9 ± 136.8	235.3 ± 130.4	245.7 ± 142.7	0.34
ASA, American Society of Anesthesiologists; ERCP, endoscopic retrograde cholangiopancreatography; EUS-CDS, endoscopic ultrasound-guided choledochoduodenostomy; MDBO, malignant distal biliary obstruction.				

**Table:** **Table 2** Pooled rates or primary and secondary endpoints using individual patient data.

**Variable**	**Overall n = 519**	**EUS-CDS (n = 263) n (%)**	**ERCP (n = 256) n (%)**	**Meta-analysis aggregate data** **Risk ratio (95%CI)** **or WMD (95%CI)** ** I ^2^ , τ ^2^ , Cochrane *P* value for heterogeneity **	**Meta-analysis** **individual patient data** **Odds ratio (95% CI) or WMD** **(95% CI)** **(n = 519 patients)**
Primary outcome					
30-day procedure related adverse events	78 (15.0%)	34 (12.9%)	44 (17.2%)	0.88 (0.60, 1.29) I ^2^ = 0%; τ ^2^ = 0; *P* = 0.66 3 studies, n=519	0.72 (0.44–1.16)
Secondary outcome					
Severe or fatal procedure related adverse events (within 14 days of the procedure)	15 (2.9%)	6 (2.3%)	9 (3.5%)	0.76 (0.31; 1.83) I ^2^ = 0%, τ ^2^ = 0; *P* = 0.64 3 studies, n = 519	0.64 (0.22–1.83)
Stent dysfunction	42 (8.1%)	19 (7.2%)	23 (9.0%)	0.86 (0.50; 1.48) I ^2^ = 0%; τ ^2^ = 0; *P* = 0.93 3 studies, n = 519	0.79 (0.42–1.50)*
Technical success	448 (86.3%)	247 (93.9%)	201 (78.5%)	1.18 (1.09; 1.28) I ^2^ = 36%, τ ^2^ = 0; *P* = 0.21 3 studies, n = 519	4.22 (2.35–7.61)*
Clinical success	441 (85.0%)	224 (85.2%)	217	1.00 (0.95; 1.07) I ^2^ = 0%, τ ^2^ = 0; p = 0.74 3 studies, n = 519	1.03 (0.64–1.67)
Procedure time	20.2 ± 16.1	15.1 ± 13.9	25.6 ± 16.5	-8.63 (-14.28; -2.98)† I ^2^ = 69%, τ ^2^ = 12; *P* = 0.07 2 studies, n = 364†	-10.50 (-15.28 to -5.73)
*Unadjusted odds ratios. †Teoh et al. reported median and ranges. The latter could not be converted to standard deviation. ERCP, endoscopic retrograde cholangiopancreatography; EUS-CDS, endoscopic ultrasound-guided choledochoduodenostomy.					

**Fig. 4:**
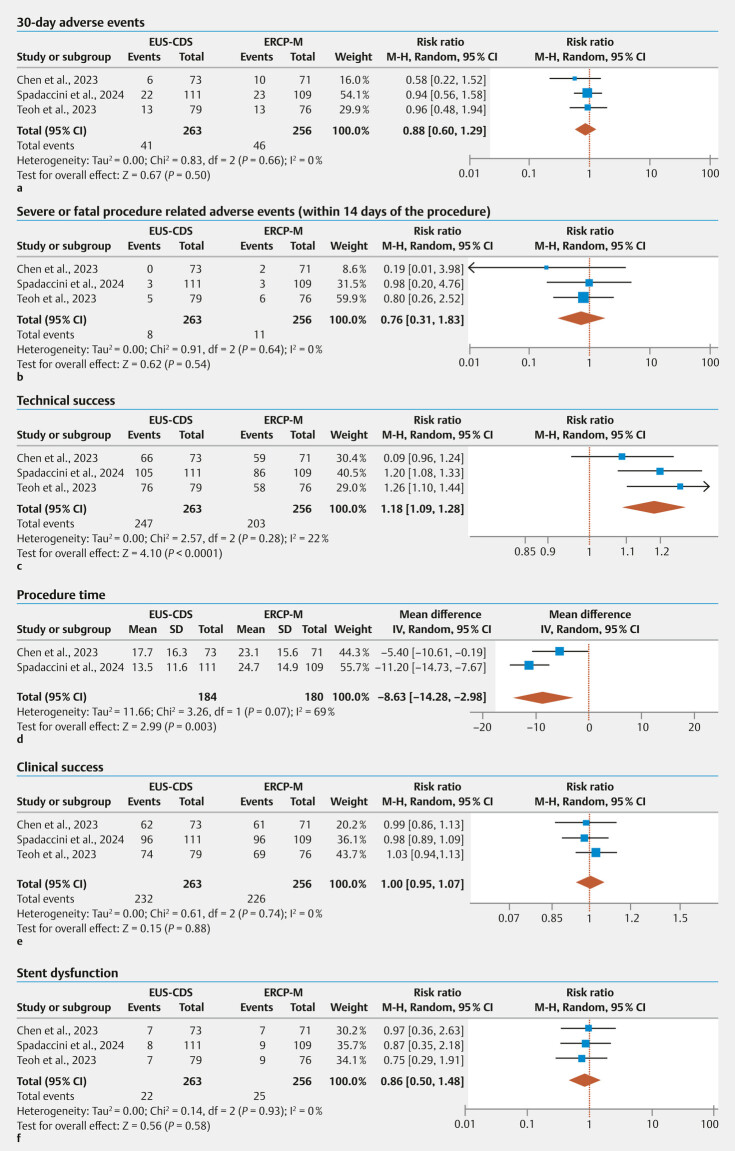
Aggregate meta-analysis. Forrest plot demonstrating the relative risk of **a** overall 30-day procedure related adverse event; **b** Severe or fatal procedure related severe adverse events; **c** technical success; **d** procedure time; **e** clinical success; and **f** stent dysfunction.

In the IPD meta-analysis, estimated OR for 30-day AEs comparing EUS-CDSL with ERCP was 0.72 (95% CI 0.44–1.16). In the aggregate meta-analysis, EUS-CDSL was associated with a similar risk of 30-day AEs (RR 0.88, 95% CI 0.60–1.29) ( Fig. 4 ). For the IPD analysis, the CI includes both a clinically meaningful reduction and a modest increase in AEs, reflecting residual sampling uncertainty; however, the small upper confidence limit likely excludes clinically relevant increases in 30-day AE risk. Using the AE event rate of 17.2% for ERCP the upper bound OR **1.16** corresponds to **+2.2% absolute difference** for EUS-CDSL.

In both the IPD and aggregate meta-analyses, no statistically significant differences were observed in 14-day procedure-related severe or fatal AEs (IPD OR 0.64, 95% CI 0.22–1.83; aggregate RR 0.76, 95% CI 0.31–1.83). Given the low event rates, these estimates, however, remain imprecise, with some residual uncertainty for both clinically meaningful benefit and harm.

The IPD meta-analysis demonstrated that technical success was higher with EUS-CDSL (OR 4.22, 95% CI 2.35–7.61). Similarly, the aggregate meta-analysis showed superior technical success with EUS-CDSL (RR 1.18, 95% CI 1.09–1.28). Procedure time was also shorter for EUS-CDSL in both the aggregate (two trials) and IPD (three trials) analyses ( Table 2 ).

Lastly, both the IPD and aggregate data meta-analyses yielded no significant differences in clinical success (OR 1.03, 95% CI 0.64–1.67 and RR 1.00, 95% CI 0.95- 1.07) or stent dysfunction (OR 0.79, 95% CI 0.42–1.50 and RR 0.86, 95% CI 0.50–1.48). The CIs for both clinical success and stent dysfunction, however, lack certainty in excluding small but clinical meaningful differences. Subgroup analysis conducted for fixed effect models provided similar results.

## Discussion

EUS-CDSL is emerging as a viable first-line alternative to ERCP in MDBO. Randomized trials have reported comparable clinical outcomes with certain technical advantages; however, individual studies have had limited precision to reliably characterize procedure-related safety 6 10 . In our IPD meta-analysis of three RCTs, we sought to more precisely evaluate safety and technical performance of EUS-CDSL compared with ERCP. We found no statistically significant difference in overall 30-day procedure-related AEs (OR 0.72; 95% CI 0.44–1.16), with lower bound CI compatible with substantial benefit and the relatively small upper bound CI suggesting low likelihood of substantial increase in harm. Severe or fatal procedure-related AEs were uncommon in both groups, although the available data remain imprecise for excluding small but clinically important differences in these rare outcomes. Technically, EUS-CDSL was associated with higher technical success and shorter procedure time. Taken together, these findings suggest that EUS-CDSL offers meaningful technical advantages over ERCP with comparable safety, while acknowledging residual uncertainty for rare severe or fatal AEs.

The advent of biliary LAMS has greatly simplified the technique of EUS-BD, which has been limited in adoption due to its technical complexity and lack of dedicated devices 23 . The cautery LAMS allows for a one-step biliary drainage using only one device without requiring needle puncture, expert guidewire manipulation, or tract dilation 7 10 . Newfound ease of technical adoption is highlighted in relative inexperience of the operators in the included trials, especially in the RCT by Chen et. al. in which the median number of EUS-CDSL performed by operators prior to entry into the trial was only two. Even in the trial by Teoh et. al., operators had to only perform 20 EUS-CDSLs to be eligible to participate in the study. These numbers are in stark contrast to the control arm of ERCP, which included only operators with more than 1000 career ERCPs. Despite the disproportionate advantage in experience in favor of the ERCP arm, our IPD meta-analysis showed that EUS-CDSL outperformed ERCP in technical success and procedure time, while having comparable risks and odds for 30-day procedure-related AEs and severe or fatal procedure-related severe AEs. One important caveat, however, is that the included trials only included patients with bile duct diameter > 12 mm 6 10 or 15 mm 21 , therefore, our findings cannot be generalized to patients with MDBO and minimally dilated bile ducts. Taken together, our data suggest that EUS-CDSL may be a technically easier and more efficient procedure than ERCP in patients with dilated bile ducts. This efficiency is likely further highlighted when EUS-guided tissue diagnosis is needed such that EUS-CDSL can be performed with the same echoendoscope instead of processing a second duodenoscope for ERCP. The decision to proceed to EUS-CDSL or ERCP in this clinical setting can then be dictated by the bile duct diameter and scope position during the diagnostic portion of the EUS. In addition, EUS-CDSL can be performed without fluoroscopic guidance, which further streamlines its use 10 .

Our aggregate and IPD meta-analyses both demonstrate comparable risks and odds for clinical success and stent dysfunction between EUS-CDSL and ERCP in MDBO; however, the somewhat imprecise CIs suggest some uncertainty toward possible clinical differences. It is important to note that only patients with unresectable peri-ampullary cancers or locally advanced/borderline resectable cancers who were not candidates for upfront resection were included. No current RCT data exist for EUS-biliary drainage in resectable patients. Although EUS-CDSL has the theoretical advantage of providing a bypass and thus preventing stent dysfunction from direct tumor tissue stent ingrowth and overgrowth, other causes of stent dysfunction may occur, including food impaction and sump syndrome 6 8 9 10 . Moreover, studies included in the meta-analyses did not consider patients with clinical evidence of malignant gastric outlet obstruction (MGOO). Given that MGOO is most often post bulbar, stent patency is likely a major problem in this setting where there is stasis of food in the duodenal bulb and inability for the bile to flow distally down the gastrointestinal tract. As such, in the setting of MGOO, EUS-CDSL likely requires concomitant endoscopic relief of MGOO either through enteral stenting or EUS-guided gastroenterostomy 24 to achieve adequate stent patency. The optimal palliation of this patient population requires further study.

The cost of LAMS has been identified as a major barrier to widespread clinical adoption of EUS-CDSL 23 . There are no cost-effectiveness data available to inform whether the greater efficiency and technical success of EUS-CDSL can offset the higher upfront cost of LAMS. This significant hurdle will likely continue to limit clinical use of EUS-CDSL, highlighting need for alternative device options to reduce costs. Once the cost of a dedicated EUS-CDSL stent achieves a cost-effectiveness profile, widespread adoption of EUS-CDSL as first-line modality in patients with MDBO will likely ensue.

Our IPD meta-analysis has certain limitations. First, our study included only three RCTs, precluding formal study-level hierarchical modeling. Although individual randomization—and center-stratified allocation in two trials—reduces likelihood of confounding by center, formal modeling of center-level clustering was not feasible because of sparse events, and residual variation related to operator or institutional factors cannot be fully excluded. Stent dysfunction was analyzed as a binary outcome rather than as a time-to-event endpoint; therefore, death prior to documentation of dysfunction was not formally modeled as a competing risk and may have led to underestimation of dysfunction rates. However, no differences in overall survival were observed between treatment arms, making differential bias from competing events unlikely. The extremely low incidence of severe or fatal AEs resulted in imprecise CIs, limiting certainty for rare safety outcomes. In addition, because outcomes such as technical success were common, ORs may overstate relative effect size; absolute differences and relative risks, therefore, should guide clinical interpretation. Finally, all included trials excluded patients with minimally dilated bile ducts, limiting generalizability to this common subgroup 25 . Strengths of our meta-analysis are inclusion of high-quality RCTs using both IPD and aggregate meta-analytical methodologies. IPD is the gold standard for meta-analysis, allowing for use a homogenous definition for each endpoint, thus enhancing precision, directness, and overall certainty of the data 26 . The multicenter design of the trials, across several continents, also increases broad generalizability of our results with regard to operator expertise, patient demographics, and health care systems.

## Conclusions

In conclusion, our IPD and aggregate meta-analyses of RCTs provide compelling evidence supporting adoption of EUS-CDSL in expert hands as a safe and efficient alternative to ERCPwhen managing patients with MDBO and significant biliary dilation who are not candidates for upfront curative surgical resection. In addition, EUS-CDSL was associated with higher technical success and shorter procedure time; however, it may not be suitable in MDBO with minimally dilated ducts or in patients with concomitant gastric outlet obstruction. Controlled data comparing EUS-CDSL to ERCP in resectable disease in addition to cost-effectiveness analysis are needed to better inform its clinical implementation.

## Supplementary Material

Supplementary Material Zusatzmaterial
