# Supplementary material for: Endosonography-guided choledochoduodenostomy using a lumen apposing metal stent vs. ERCP: Individual patient data and aggregate meta-analyses
Source: Endosc Int Open. 2026 Jun 1;14:a28631621. doi: 10.1055/a-2863-1621 (PMC13289975; doi:10.1055/a-2863-1621)
Supplement: Supplementary file 1 — Supplementary Material [file 10-1055-a-2863-1621_28644016.pdf]

Supplementary Table 1 Search string.

EMBASE (Cochrane Highly Sensitive Search Strategy for identifying controlled trials in Embase)[24]
(Randomized controlled trial/ or Controlled clinical study/ or random*.ti,ab. or randomization/ or intermethod comparison/ or placebo.ti,ab. or (compare or compared or comparison).ti. or ((evaluated or evaluate or evaluating or assessed or assess) and (compare or compared or comparing or comparison)).ab. or (open adj1 label).ti,ab. or ((double or single or doubly or singly) adj1 (blind or blinded or blindly)).ti,ab. or double blind procedure/ or parallel group*1.ti,ab. or (crossover or cross over).ti,ab. or ((assign* or match or matched or allocation) adj5 (alternate or group*1 or intervention*1 or patient*1 or subject*1 or participant*1)).ti,ab. or (assigned or allocated).ti,ab. or (controlled adj7 (study or design or trial)).ti,ab. or (volunteer or volunteers).ti,ab. or human experiment/ or trial.ti.) not (((random* adj sampl* adj7 ("cross section*" or questionnaire*1 or survey* or database*1)).ti,ab. not (comparative study/ or controlled study/ or randomized controlled.ti,ab. or randomly assigned.ti,ab.)) or (Cross-sectional study/ not (randomized controlled trial/ or controlled clinical study/ or controlled study/ or randomized controlled.ti,ab. or control group*1.ti,ab.)) or (((case adj control*) and random*) not randomized controlled).ti,ab. or (Systematic review not (trial or study)).ti. or (nonrandom* not random*).ti,ab. or "Random field*".ti,ab. or (random cluster adj3 sampl*).ti,ab. or ((review.ab. and review.pt.) not trial.ti.) or ("we searched".ab. and (review.ti. or review.pt.)) or "update review".ab. or (databases adj4 searched).ab. or ((rat or rats or mouse or mice or swine or porcine or murine or sheep or lambs or pigs or piglets or rabbit or rabbits or cat or cats or dog or dogs or cattle or bovine or monkey or monkeys or trout or marmoset*1).ti. and animal experiment/) or (Animal experiment/ not (human experiment/ or human/)))
AND
(Pancreas/ OR pancreas.ti,ab.) AND (neoplasms/ OR tumor.mp. OR cancer.mp. OR malignan$.mp. OR adenocarcinoma/ or adenocarcinoma.mp.) OR pancreas adenocarcinoma/ OR Bile Duct Neoplasms/ or (Biliary adj1 Obstruction).mp.
AND
Cholangiopancreatography, Endoscopic Retrograde/ OR Self Expandable Metallic Stents/ OR (EUS-guided adj1 biliary adj1 drainage).mp. OR (choledochoduodenostomy).mp. OR (endoscopic aj1 ultrasound aj1 guided).mp. OR LAMS.mp. OR Axios.mp. OR Spaxus.mp. OR (Nagi adj1 stent).mp.

Medline (Cochrane Highly Sensitive Search Strategy for identifying randomized trials in MEDLINE:)[24]
(randomized controlled trial or controlled clinical trial).pt. or randomized.ab. or randomised.ab. or placebo.ab. or drug therapy.fs. or randomly.ab. or trial.ab. or groups.ab.) not (exp animals/ not humans.sh.)
AND
(Pancreas/ OR pancreas.ti,ab.) AND (neoplasms/ OR tumor.tw. OR cancer.mp. OR malignan$.tw. OR adenocarcinoma/ or adenocarcinoma.tw.) OR pancreas adenocarcinoma/

ISI Web of Science:
(randomized AND controlled AND trial*) OR (controlled AND clinical AND trial*) OR (random AND allocation) OR (random* AND allocated) OR (double AND blind AND method) OR (single AND blind AND method)
AND

Pancreas AND (neoplasms OR tumor OR cancer OR malignan$ OR adenocarcinoma or adenocarcinoma) OR pancreas adenocarcinoma and (Biliary and Obstruction)
AND
Cholangiopancreatography, Endoscopic Retrograde/ OR Self Expandable Metallic Stents/ OR (EUS-guided adj1 biliary adj1 drainage).mp. OR (choledochoduodenostomy).mp. OR (endoscopic aj1 ultrasound aj1 guided).mp. OR LAMS.mp. OR Axios.mp. OR Spaxus.mp. OR (Nagi adj1 stent).mp.

Supplementary Table 2

Certainty assessment							Summary of findings				
Participants (studies) Follow-up	Risk of bias	Inconsistency	Indirectness	Imprecision	Publication bias	Overall certainty of evidence	Study event rates (%)		Relative effect (95% CI)	Anticipated absolute effects	
							With [comparison]	With [intervention]		Risk with [comparison]	Risk difference with [intervention]
**All 30-day adverse events**											
519 (3 RCTs)	not serious	not serious	not serious	serious[a]	none	⊕⊕⊕◯ Moderate[a]	46/256 (18.0%)	41/263 (15.6%)	**RR 0.88** (0.60 to 1.29)	46/256 (18.0%)	**22 fewer per 1,000** (from 72 fewer to 52 more)
**Severe or fatal procedure related adverse events (within 14 days of the procedure)**											
519 (3 RCTs)	not serious	not serious	not serious	serious[a]	none	⊕⊕⊕◯ Moderate[a]	11/256 (4.3%)	8/263 (3.0%)	**RR 0.76** (0.31 to 1.83)	11/256 (4.3%)	**10 fewer per 1,000** (from 30 fewer to 36 more)
**Technical success**											
519 (3 RCTs)	not serious	not serious	not serious	serious[a]	none	⊕⊕⊕◯ Moderate[a]	203/256 (79.3%)	247/263 (93.9%)	**RR 1.18** (1.09 to 1.28)	203/256 (79.3%)	**143 more per 1,000** (from 71 more to 222 more)
**Procedure time**											
364 (2 RCTs)	serious[b]	not serious	not serious	serious[a]	none	⊕⊕◯◯ Low[a,b]	180	184	-	180	MD **8.63 SD lower** (14.28 lower to 2.98 lower)
**Clinical success**											
519 (3 RCTs)	not serious	not serious	not serious	serious[a]	none	⊕⊕⊕◯ Moderate[a]	232/256 (90.6%)	224/263 (85.2%)	**RR 1.00** (0.95 to 1.07)	232/256 (90.6%)	**0 fewer per 1,000** (from 45 fewer to 63 more)
**Stent dysfunction**											

Supplementary Table 2

Certainty assessment							Summary of findings				
519<br>(3 RCTs)	not serious	not serious	not serious	serious[a]	none	⊕⊕⊕◯<br>Moderate[a]	25/256 (9.8%)	22/263 (8.4%)	**RR 0.86**<br>(0.50 to 1.48)	25/256 (9.8%)	**14 fewer per 1,000**<br>(from 49 fewer to 47 more)

CI, confidence interval; MD, mean difference; RR, risk rati.o

Explanations

a. Small sample size, wide confidence interval.

b. statistical heterogeneity.
